# Effect of the SARS-CoV-2 Pandemic on Gastrointestinal Endoscopy Procedures: Experience of a Tertiary Care Center From Eastern India

**DOI:** 10.7759/cureus.41315

**Published:** 2023-07-03

**Authors:** Ravikant Kumar, Sanjeev K Jha, Saumyaleen Roy, Vishwa M Dayal

**Affiliations:** 1 Gastroenterology, Indira Gandhi Institute of Medical Sciences, Patna, IND

**Keywords:** sars-cov-2, safety, rt-pcr, lockdown, ercp, endoscopy, covid-19, colonoscopy

## Abstract

Background

To contain the spread of infection and reduce the burden on healthcare infrastructure, many countries globally adopted a lockdown strategy during the severe acute respiratory syndrome coronavirus 2 (SARS-CoV-2) pandemic. Hospitals were converted to dedicated coronavirus disease 2019 (COVID-19) centers, and non-COVID-19 patients were intervened on a triage basis. During this time, only emergency procedures were performed. The impact of this lockdown strategy during the first wave of the SARS-CoV-2 pandemic on various gastrointestinal endoscopy interventions remains unknown.

Methodology

In this retrospective, observational study conducted in the Department of Gastroenterology, Indira Gandhi Institute of Medical Sciences, Patna, Bihar from March 25 to September 30, 2020, data related to clinical profile, indication, and endoscopic interventions performed in reverse transcriptase-polymerase chain reaction (RT-PCR)-negative patients with the use of personal protective kits were analyzed and compared with the historical controls.

Results

A total of 2,282 patients were admitted and 422 endoscopic procedures were performed during the six-month study period with an intervention rate of 18.49%. The most common procedure was upper gastrointestinal endoscopy (228, 58.13%), followed by endoscopic retrograde cholangiopancreatography (ERCP) (88, 22.50%). Chronic liver disease (CLD) (144 patients) followed by malignancy (111 patients) were the most common diagnosis. During the first phase of the lockdown (March to May), only 52 procedures were performed (52 vs. 506). None of the patients underwent endoscopic ultrasound. In 2019, 4,501 patients were admitted and 1,224 procedures were performed with an intervention rate of 27.19 (p < 0.0001). None of the staff of the Department of Gastroenterology developed symptomatic SARS-CoV-2 infection during this period.

Conclusions

There was a significant drop in endoscopic procedures during the lockdown and most of the esophagoduodenoscopy procedures were done for CLD and ERCP for biliary tract malignancy. Endoscopic procedures done on RT-PCR for COVID-19-negative patients were safe using personal protective kits.

## Introduction

Coronavirus disease 2019 (COVID-19) is caused by a novel coronavirus (nCoV-19) called severe acute respiratory syndrome coronavirus 2 (SARS-CoV-2). SARS-CoV-2 was first described in Wuhan, a city in the Hubei Province of China, which led to a cluster of pneumonia cases at the end of 2019 [1]. The high infective rate of SARS-CoV-2 has disrupted lives, livelihoods, communities, and businesses worldwide. The World Health Organization (WHO) declared this a global health emergency at the end of January 2020. India reported its first confirmed case of COVID-19 on January 30, 2020, from Kerala, and it spread rapidly to every state and union territory of the country leading to a huge burden on the healthcare infrastructure. About 60% of COVID-19 transmission is from asymptomatic individuals [2]. A secondary attack rate of up to 15% was seen in household contacts [3-7]. However, many individuals with COVID-19 do not report having had specific close contact with COVID-19 in the weeks before their diagnosis [8]. Therapeutic strategies to deal with the infection are only supportive, with prevention aiming at reducing transmission in the community as the best weapon. At that time, the only way to contain the infection was the use of masks and maintaining social distancing.

COVID-19 has also affected the natural course of various diseases. It is known to cause more case fatalities in individuals with comorbidities. Hypertension [9] and diabetes [10] are among the two comorbidities which led to the highest case fatality among COVID-19 cases. At the moment, the diagnostic tests are reverse transcriptase-polymerase chain reaction (RT-PCR) and rapid antigen test with 70% and 50% sensitivity, respectively [11]. Aggressive isolation measures have led to a progressive reduction in cases. For stopping the spread of the infection, many countries imposed a complete lockdown. India also declared a complete lockdown on March 23, 2020. Hospitals were converted to dedicated COVID-19 centers which became a boon to curb infection and properly manage COVID-19 patients. However, it also caused several problems for non-COVID-19 patients to avail healthcare facilities due to transportation issues and limited non-COVID-19 beds available in hospitals.

To maintain the standards of healthcare in non-COVID-19 patients keeping in mind the risk of transmission of the virus, various societies have laid down guidelines regarding the nature of the procedure and in whom it can be performed during the pandemic. According to the Asia Pacific Society for Digestive Endoscopy [12] guidelines for endoscopy, proper triage and assessment of the risk of patients with suspected or confirmed COVID-19 before endoscopy are essential. Deferment of elective endoscopies should be considered during the COVID-19 outbreak. Urgent endoscopies should be performed by strategically assigned staff to minimize concomitant exposure and proper personal protective equipment (PPE) kit should be provided to the endoscopist.

As Indira Gandhi Institute of Medical Sciences (IGIMS) is the only public sector tertiary care center in Bihar rendering super-specialty services, it was crucial to perform endoscopic procedures abiding by COVID-19 guidelines during the lockdown period. However, the impact of this lockdown and triage strategy for performing endoscopic procedures is not well described. Hence, we aimed to evaluate the clinical profile, indication, and type of endoscopic procedures performed during the first wave of the COVID-19 pandemic and compared it with the historical control.

This article was previously presented as a poster at the 62nd annual conference of the Indian Society of Gastroenterology on February 10-13, 2022, in Pune.

## Materials and methods

This retrospective study was conducted in the Department of Gastroenterology, IGIMS, Patna. Patients undergoing gastrointestinal endoscopic procedures during the lockdown period (March 25, 2020, to September 30, 2020) were enrolled and their clinical profiles, indications for endoscopy, and outcomes were analyzed. The clinical profile of the cases was obtained from the indoor register, and the details of endoscopic procedures were retrieved from the endoscopic diary. All endoscopic procedures were performed within five days of a negative RT-PCR report for COVID-19. Any patient who tested positive for COVID-19 during the hospital stay or was COVID-19 positive at the time of admission was excluded and referred to a dedicated COVID-19 hospital. Endoscopists and staff of the endoscopy room used full PPE kits (N-95 mask, face shield, goggles, hand gloves, coveralls, and shoe covers). Standard sterilization procedures for endoscopes and accessories were performed as usual. There was no provision for a negative-pressure endoscopy room. All data were compared with data from the last year during the same time interval to determine the impact of the COVID-19 pandemic and the nationwide lockdown strategy.

Numerical variables were expressed as mean ± SD and median (range). Comparison of parametric data was done using a two-sample t-test, and for the non-parametric data, the Mann-Whitney test was used. A p-value below 0.05 was considered statistically significant.

The study was approved by the Institutional Ethics Committee of Indira Gandhi Institute of Medical Sciences (approval number: 1919/IEC/IGIMS/2020) and was conducted in accordance with the principles of the Declaration of Helsinki as revised in 2000. As it was a retrospective analysis of data retrieved from indoor patients and endoscopy diary records, consent was exempted.

## Results

**Figure 1:**
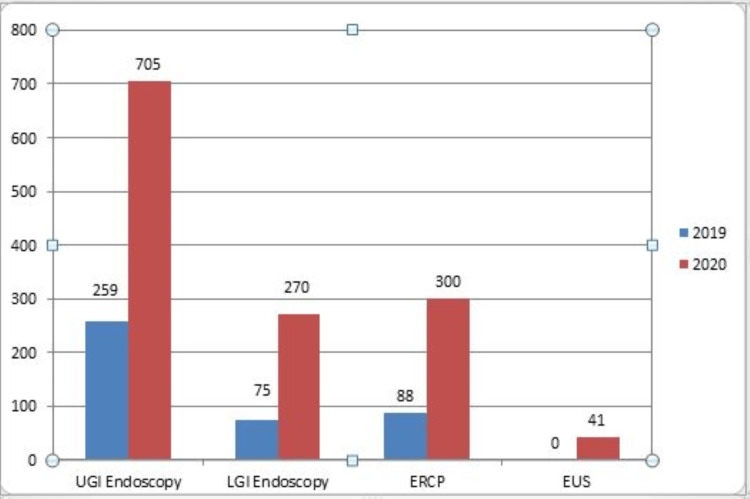
Bar diagram showing various endoscopic procedures conducted in 2019 and 2020. UGI = upper gastrointestinal tract; LGI = lower gastrointestinal tract; ERCP = endoscopic retrograde cholangiopancreatography; EUS = endoscopic ultrasound

**Figure 2:**
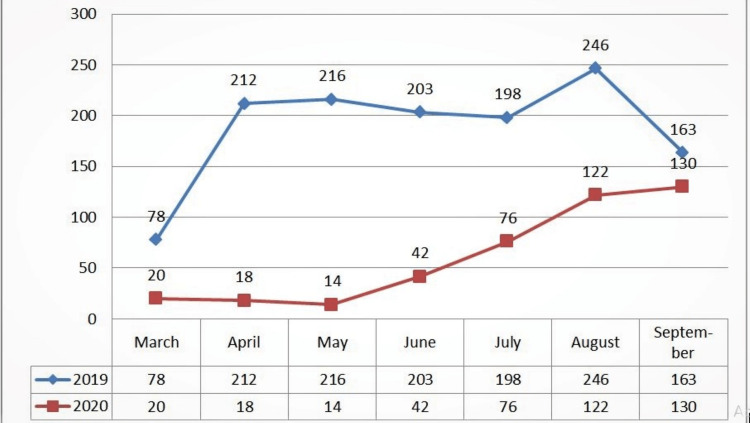
Monthly split of procedures performed between March and September in 2020 and 2019.

Out of 2,282 patients, 422 endoscopic procedures were performed on 391 patients during the six-month study period (March 25th to September 30th, 2020) with an intervention rate of 18.49%. The most common procedure was upper gastrointestinal endoscopy (UGIE) (259, 61.37%), followed by endoscopic retrograde cholangiopancreatography (ERCP) (88, 20.85%) (Table 1). Overall, 75 (17.77%) patients underwent colonoscopy or sigmoidoscopy (LGIE). During this period in 2019, 1,316 endoscopic procedures were performed on 1,175 patients (Figure 1) with an intervention rate of 32.89%. Out of 1,316 procedures performed in the same six months of 2019, 705 patients underwent UGIE (53.57%), 270 patients underwent LGIE (20.51%), 300 patients underwent ERCP (22.79%), and 41 patients underwent endoscopic ultrasound (EUS) (3.11%). There were very few procedures performed in March, April, and May 2020 and the number started to increase from June, with procedures performed during September 2020 being comparable to those performed in September 2019 (120 vs. 113) (Figure 2).

**Table 1:** Indications for endoscopic procedures performed from March to September in 2019 and 2020.

Indication for endoscopy	Number of patients	
Year	2020	2019
Dyspepsia	60	226
Dysphagia	10	38
Hematemesis	23	30
Melena	30	98
Variceal screening	96	175
Gastric outlet obstruction	10	36
Hematochezia	49	200
Diarrhea	26	80
Cholangitis with jaundice	38	89
Cholestatic jaundice without cholangitis	49	203

Table 2 and Table 3 show the clinical presentation and diagnosis of patients undergoing endoscopic procedures during the study period of 2020 and 2019. The most common diagnosis for patients undergoing UGIE was chronic liver disease (CLD) who presented with either hematemesis and/or melena or screening for high-risk varices. Out of 88 ERCPs performed during the lockdown, 60 (68.18%) were for malignant biliary tract diseases and 28 (31.82%) for cholangitis. Choledocholithiasis (127 patients, 42.33%) was the most common indication for ERCP in 2019.

**Table 2:** Clinical profile of patients who were intervened in 2019 and 2020. IBD = inflammatory bowel disease; CBD = common bile duct

Clinical profile of patients	Number of patients	
Year	2020	2019
Normal study	24	50
Chronic liver disease	144	273
Acid peptic disorder	52	256
Hemorrhoids	15	102
Nonspecific caecal ulceration	9	10
IBD	8	60
Carcinoma esophagus	4	30
Carcinoma stomach	24	36
Carcinoma colon	23	58
CBD stone	28	127
CBD stricture	0	42
CBD injury	0	16
Periampullary carcinoma	26	43
Cholangiocarcinoma	12	23
Gallbladder malignancy	22	49

**Table 3:** Comparison of endoscopic procedures performed from March to September in 2019 and 2020. *: chi-square test; †: Fisher exact test. UGI = upper gastrointestinal tract; ERCP = endoscopic retrograde cholangiopancreatography; EUS = endoscopic ultrasound

Procedures	2020	2019	P-value
UGI endoscopy	259	705	<0.0001^*^
Colonoscopy	75	270	<0.0001^*^
ERCP	88	300	<0.0001^*^
ERCP for benign disease	28	185	<0.0001^*^
ERCP for malignant disease	60	115	<0.0001^*^
EUS	0	41	<0.0001^†^

## Discussion

All endoscopic procedures, including UGIE, EUS, and ERCP, have the potential for increased generation of infected droplets during coughing, retching, and suctioning, creating aerosolization and increased risk of transmission [13,14]. Endoscopy centers usually deal with high-volume and close-contact procedures which can make them high-risk COVID-19 transmission areas if extreme precautions are not taken [15]. Due to concern regarding the spread of infection, all elective endoscopic procedures were halted during the first phase of the lockdown, and only emergency procedures were performed on RT-PCR-confirmed COVID-19-negative patients. All procedures were performed following the guidelines of the Asia Pacific Society for Digestive Endoscopy. Use of PPE kits and testing for nCoV-19 can reduce stress and anxiety in healthcare professionals [16-19].

There was a substantial reduction in the number of endoscopic procedures performed in IGIMS during the six-month lockdown period compared to last year. A total of 1,316 procedures were performed in 2019 (March 25 to September 30) compared to 422 procedures performed in 2020 with a 68% reduction (p < 0.0001). Out of 1,316 procedures performed (from March to September) in 2019, 705 (53.57%) patients underwent UGIE, 270 (20.51%) patients underwent LGIE, 300 (22.79%) patients underwent ERCP, and 41 (3.11%) patients underwent EUS. In 2020, out of the 422 procedures performed on 391 patients (from March to September), 259 (61.37%) patients underwent UGIE, 75 (17.77%) patients underwent LGIE, 88 (20.85%) patients underwent ERCP, and none of the patients underwent EUS. During the six-month lockdown phase, there was a 63% reduction noted in UGIE, a 72.23% reduction in LGIE, and a 70.66% reduction in ERCP procedures from 2019.

During the first phase of the lockdown (25th March to 31st May), only 41 endoscopic procedures were performed compared to the last year when 506 procedures were performed during the same period. Due to transportation issues, strict lockdown guidelines, and screening out elective cases, the endoscopic procedures showed a significant decline. All procedures performed were emergent such as upper gastrointestinal, lower gastrointestinal bleeding, and cholangitis.

Once the lockdown was lifted in a staged manner, the patient load started to increase. During the first unlock period (June 1st to June 30th), 42 endoscopic procedures were performed and all were emergency procedures compared to the last year when 203 procedures were performed. The emergency procedures were still on the lower side compared to previous years as people had fear of contracting COVID-19 during traveling, inadequate maintenance of social distancing in hospital premises, and transportation issues.

During the second unlock period, the number of patients started increasing in the outpatient department, and emergency elective procedures were started as the number of backlog cases increased. Patients were given dates for elective procedures and emergency procedures were performed on an urgent basis. In total, 76 procedures were performed, of which 32 were performed electively.

During the third and fourth unlock periods, elective cases also showed increasing trends. In the month of September, the total number of cases performed outnumbered the number of procedures done in the last year, as, from the month of September, routine endoscopic cases were regularly performed after RT-PCR testing for COVID-19 done on an outpatient basis. The rise in elective cases compared to last year is probably due to the centers in periphery not performing elective cases. The reported sensitivity for RT-PCR is 63-78% [20]. This medium sensitivity of RT-PCR creates a sense of fear in healthcare workers for dealing with any type of patient in the COVID-19 era. Although there were no facilities for a negative-pressure room, none of our endoscopy staff contracted the disease. None of the patients who underwent endoscopic procedures developed COVID-19 symptoms during the hospital stay. Hence, we did not see any cross-infection in our study. These may be attributed to the low viral load, low infectivity of the first-wave strain, and low infectivity of asymptomatic patients [21,22]. As of now, most healthcare workers are vaccinated, and the safety of endoscopic procedures on RT-PCR-negative patients with the proper use of PPE kits will alleviate the fear of contracting infection among healthcare workers, with endoscopic facilities being available more easily to patients in this pandemic era.

Our study has some limitations. As this is a retrospective single-center study, the findings need to be further investigated. Infectivity and virulence of the virus depend on the strain which was not ascertained in this study. Asymptomatic infections are common in SARS-CoV-2, and RT-PCR was done on healthcare workers presenting with symptoms of respiratory tract infection.

## Conclusions

The COVID-19 pandemic has placed a substantial toll on the healthcare system and significant impact on gastrointestinal endoscopic procedures. During the first phase of the COVID-19 pandemic, very few endoscopic procedures were performed in selected cases. Gastrointestinal endoscopy is safe using PPE kits in patients whose RT-PCR for COVID-19 is negative. However, it needs further research taking into account factors such as viral strain.
